# Non-classical roles of bacterial siderophores in pathogenesis

**DOI:** 10.3389/fcimb.2024.1465719

**Published:** 2024-09-20

**Authors:** Elliot Arnold

**Affiliations:** Department of Biosciences, Durham University, Durham, United Kingdom

**Keywords:** siderophores, virulence factors, bacterial pathogenesis, signalling, reactive oxygen species, metals, microcins

## Abstract

Within host environments, iron availability is limited, which instigates competition for this essential trace element. In response, bacteria produce siderophores, secondary metabolites that scavenge iron and deliver it to bacterial cells via specific receptors. This role in iron acquisition contributes significantly to bacterial pathogenesis, thereby designating siderophores as virulence factors. While prior research has primarily focused on unravelling the molecular mechanisms underlying siderophore biosynthesis, uptake, and iron sequestration, recent investigations have unveiled additional non-iron chelating functions of siderophores. These emerging roles are being consistently shown to support bacterial pathogenesis. In this review, we present the current understanding of siderophores in various roles: acquiring non-iron metal ions, supporting tolerance to metal-induced and reactive oxygen species (ROS)-induced stresses, mediating siderophore signalling, inducing ROS formation, and functioning in class IIb microcins. By integrating recent findings, this review aims to provide an overview of the diverse roles of siderophores in bacterial pathogenesis.

## Introduction

Iron is essential for most microorganisms. Yet, despite being the fourth most abundant element in the Earth’s crust, iron is not readily available to bacteria. This is due to the oxygen-rich atmosphere oxidising soluble ferrous iron (Fe^2+^) to insoluble ferric iron (Fe^3+^) in neutral and basic pH levels. As bacteria require iron in concentrations far exceeding those available in their environment, microbes have evolved mechanisms to acquire iron, including siderophores. These low molecular weight secondary metabolites form soluble siderophore-Fe^3+^ complexes to scavenge iron before active uptake via specific receptors. As most bacteria produce siderophores, these chelators have become increasingly chemically diverse (Hider and Kong, 2010). Despite this variability, siderophores are classified into catecholate, phenolate, carboxylate and hydroxamate, depending upon the moiety of the oxygen ligands used for Fe^3+^ coordination (Raymond and Dertz, 2004).

Siderophores are well-established virulence factors due to their ability to acquire iron from the host (Bullen et al., 1999), and are implicated in the virulence of many pathogenic bacteria, including *Pseudomonas aeruginosa* (Meyer et al., 1996); *Staphylococcus aureus* (Dale et al., 2004); *Yersinia pestis* (Fetherston et al., 2010); uropathogenic *Escherichia coli* (UPEC; Torres et al., 2001); *Staphylococcus epidermidis* (Oliveira et al., 2021) and *Klebsiella pneumoniae* (Russo et al., 2014). However, it is now recognised that siderophores have more complex roles in pathogenesis. While the non-classical roles of siderophores have been excellently reviewed previously (Johnstone and Nolan, 2015), we review recent research that has significantly expanded the non-classical roles of siderophores in supporting bacterial pathogenesis (**Table 1**).

**Table 1:** Siderophores and their non-classical functions.

Siderophore	Type	Non-classical Function(s)	Reference(s)
^1^Desferrioxamine	Hydroxamate	Pro-inflammatory	Autenrieth et al. (1991) Choi et al. (2004)
		Metallophore	Evers et al. (1989)
DHBS_3_	Catecholate	Oxidative Stress Response	Bogomolnaya et al. (2020)
Enterobactin	Catecholate	Cell Signalling	Anderson and Armstrong (2004) Brickman and Armstrong (2009)
		Pro-inflammatory	Nelson et al. (2007)
		Oxidative Stress Response	Adler et al. (2012, 2014) Achard et al. (2013) Peralta et al. (2016, 2022)
Pyochelin	Phenolate	Cell Signalling	Michel et al. (2005, 2007)
		Metallophore	Braud et al. (2009a, 2010)
		Oxidative Stress-Inducing	Coffman et al. (1990) Britigan et al. (1994, 1997) Adler et al. (2012) Ong et al. (2017)
		Toxic Metal Sequestering	Braud et al. (2010)
Pyoverdine	^3^Mixed type	Cell Signalling	Lamont et al. (2002) Dumas et al. (2013) Mridha and Kümmerli (2022)
		Metallophore	Braud et al. (2009b)
		Oxidative Stress Response	Jin et al. (2018)
		Toxic Metal Sequestering	Braud et al. (2010) Lear et al. (2022)
^2^Staphyloferrin	Carboxylate	Oxidative Stress Response	Nobre and Saraiva (2014) Oliveira et al. (2021)
Yersiniabactin	Phenolate	Cell Signalling	Katumba et al. (2022) Heffernan et al. (2024)
		Metallophore	Bobrov et al. (2014, 2017) Koh et al. (2017) Robinson et al. (2018) Price et al. (2021) Behnsen et al. (2021)
		Toxic Metal Response	Chaturvedi et al. (2012)
		Oxidative Stress Response	Paauw et al. (2009) Chaturvedi et al. (2014)

^1^desferrioxamine B and E. ^2^Staphyloferrin A and B. ^3^Catecholate and hydroxamate. DHBS, 2,3-Dihydroxybenzoylserine.

## Siderophores and toxic metal tolerance

Some metals are toxic to bacteria; however, even useful metals, such as iron, zinc, and copper, become toxic at high concentrations. The immune system exploits this; for instance, neutrophils deliver zinc and macrophages deliver copper in toxic levels to the phagosome of phagocytosed bacteria (Stafford et al., 2013; Ong et al., 2014). Although the primary role of siderophores is to increase Fe^3+^ bioavailability, the structure of siderophores prevents them from being selective to one metal ion. Previously Braud et al (2009a, b). determined the wide diversity of non-ferric metal ion binding of the siderophores pyochelin and pyoverdine. In addition, Braud et al. (2010) determined that Cd^2+^, Co^2+^, Cu^2+^, Ga^3+^, Ni^2+^, TI^+^ and Zn^2+^ were more toxic in a pyochelin- and pyoverdine-deficient *P. aeruginosa* strain with both siderophores supporting resistance to metal-induced toxicity.

Expanding from this *in vitro* work, Lear et al. (2022) linked siderophore-based heavy metal detoxification and virulence *in vivo*, using the *Galleria mellonella* virulence model. They found that pyoverdine-producing *P. aeruginosa* virulence increased in copper-rich conditions, while the non-pyoverdine-producing strain virulence decreased. They suggested pyoverdine contributes to copper tolerance, which increases virulence; however, further study is required to determine the extent of siderophore-mediated protection in virulence. Additionally, as Lear et al. (2022) used a pyochelin- and pyoverdine-deficient strain to study pyoverdine’s effect on virulence in copper-replete conditions, it would be interesting to study the individual effects of pyochelin and pyoverdine on copper tolerance *in vivo.* Interestingly, pyochelin protects *P. aeruginosa* in high copper conditions *in vitro* (Braud et al., 2009a, 2010); however, both copper and pyoverdine suppress pyochelin production (Teitzel et al., 2006; Dumas et al., 2013), hinting that pyochelin may not be involved in the copper stress response. Finally, copper increased pyoverdine gene expression (Braud et al., 2009b; Lear et al., 2022), suggesting an evolved trait that links pyoverdine and copper. Unlike other siderophores, pyoverdine is not linked with copper acquisition (Braud et al., 2009b), which suggests pyoverdine increases in copper-rich conditions for copper tolerance. Also, this poses the question of how copper regulates pyoverdine synthesis.

Beyond pyoverdine, the phenolate siderophore yersiniabactin binds Cu^2+^ to prevent catechol-mediated reduction to Cu^+^, reducing copper-mediated toxicity. Notably, yersiniabactin-producing urinary isolates had significantly greater copper tolerance compared to non-producing isolates (Chaturvedi et al., 2012). Opposingly, catecholate siderophores, such as enterobactin (which is commonly co-expressed with yersiniabactin in UPEC), can reduce Cu^2+^ to Cu^+^, increasing copper toxicity (Chaturvedi et al., 2012). Interestingly, this ability of enterobactin increases the sensitivity of *E. coli* to copper. However, the CueO multicopper oxidase recovered the sensitivity of *E. coli* by oxidising enterobactin to prevent the catechol-mediated reduction of Cu^2+^ (Grass et al., 2004). Furthermore, *E. coli* strains that produced enterobactin but not enterobactin uptake and hydrolysis proteins had increased copper sensitivity and reactive oxygen species (ROS) levels compared to wild-type (Peralta et al., 2022). This research demonstrates the importance of enterobactin hydrolysis (and hints at the importance of the co-expression of yersiniabactin and enterobactin) for the protection of *E. coli* from copper toxicity. Notably, although this role of yersiniabactin has not been directly linked to enhanced pathogenesis, its importance is likely given the copper toxicity pathogenic bacteria face in the host.

## Siderophores and non-iron metal ion acquisition

The ability of siderophores to bind multiple metal ions has led to research exploring their role in acquiring metals like copper, zinc, and nickel.

First, *Y. pestis* acquires zinc through the Zn^2+^ ABC transporter ZnuABC, like other pathogens. However, a *Y. pestis* Δ*znuABC* mutant retained its virulence, suggesting an alternative mechanism of zinc acquisition (Desrosiers et al., 2010). Notably, a siderophore secreted by *Pseudomonas putida* scavenges zinc (Cortese et al., 2002; Leach et al., 2007), and pyochelin, structurally similar to yersiniabactin, binds multiple metals (Braud et al., 2009a). From this, Bobrov et al. (2014) investigated the potential of yersiniabactin in zinc acquisition. Unlike the Δ*znuABC* mutant, the Δ*irp2*Δ*znuABC* (incapable of producing yersiniabactin and ZnuABC) was attenuated in zinc-depleted media and a mouse model of septicaemic plague, suggesting yersiniabactin-mediated zinc acquisition compensates for the absent zinc transporter (Bobrov et al., 2014).

More recently, Behnsen et al. (2021) performed research to show that yersiniabactin facilitates zinc uptake in *E. coli* Nissle 1917 (EcN), aiding its colonisation of the inflamed gut where zinc is scarce due to the high expression of the zinc-chelator calprotectin (Corbin et al., 2008; Behnsen et al., 2014). Behnsen et al. (2021) showed that EcN lacking both the ZnuABC zinc transporter and ZupT zinc permease significantly outcompete EcN lacking ZnuABC, ZupT and yersiniabactin in calprotectin-supplemented media and the zinc-limited inflamed mouse gut. Significantly, beyond previous research, yersiniabactin was confirmed to bind zinc using native electrospray metabolomics. Furthermore, yersiniabactin preferentially binds iron at pH 4, zinc at pH 10, and zinc and iron equally at pH 7, potentially enhancing the ecological effectiveness of yersiniabactin. Beyond this research on probiotic EcN, Price et al. (2021) demonstrated that yersiniabactin supports *Y. pestis* pathogenesis in the zinc-limited flea midgut, highlighting it as a zinc-acquiring virulence factor. Interestingly, siderophores have been noted as pro-inflammatory (Choi et al., 2004; Nelson et al., 2007; Holden et al., 2016); therefore, whether siderophores promote colonisation through inducing inflammation-associated metal ion scarcity is noteworthy.

As yersiniabactin binds copper (Chaturvedi et al., 2012; Koh et al., 2015) and UPEC lacks copper import systems, Koh et al. (2017) hypothesised that UPEC may be able to import yersiniabactin-Cu^2+^. The group used mass spectrometry to determine yersiniabactin-Cu^2+^ forms in low-copper conditions and ^64^Cu radiolabelling to show the yersiniabactin-Cu^2+^ complex is imported via the FyuA-YbtPQ import system for utilisation in cuproenzymes. In addition, Robinson et al. (2018) used quantitative mass spectrometry to identify yersiniabactin forms yersiniabactin-Ni^2+^ complexes, which are imported for utilisation in nickel enzymes. Although yersiniabactin-mediated copper and nickel acquisition are not directly linked to virulence, they likely aid the survivability of UPEC in metal-depleted conditions. Furthermore, copper, zinc, and nickel acquisition by yersiniabactin likely contributes to its classification as a virulence factor in numerous pathogens (Koczura and Kaznowski, 2003b, 2003; Lawlor et al., 2007; Fetherston et al., 2010).

## Siderophores and signalling

**Figure 1:**
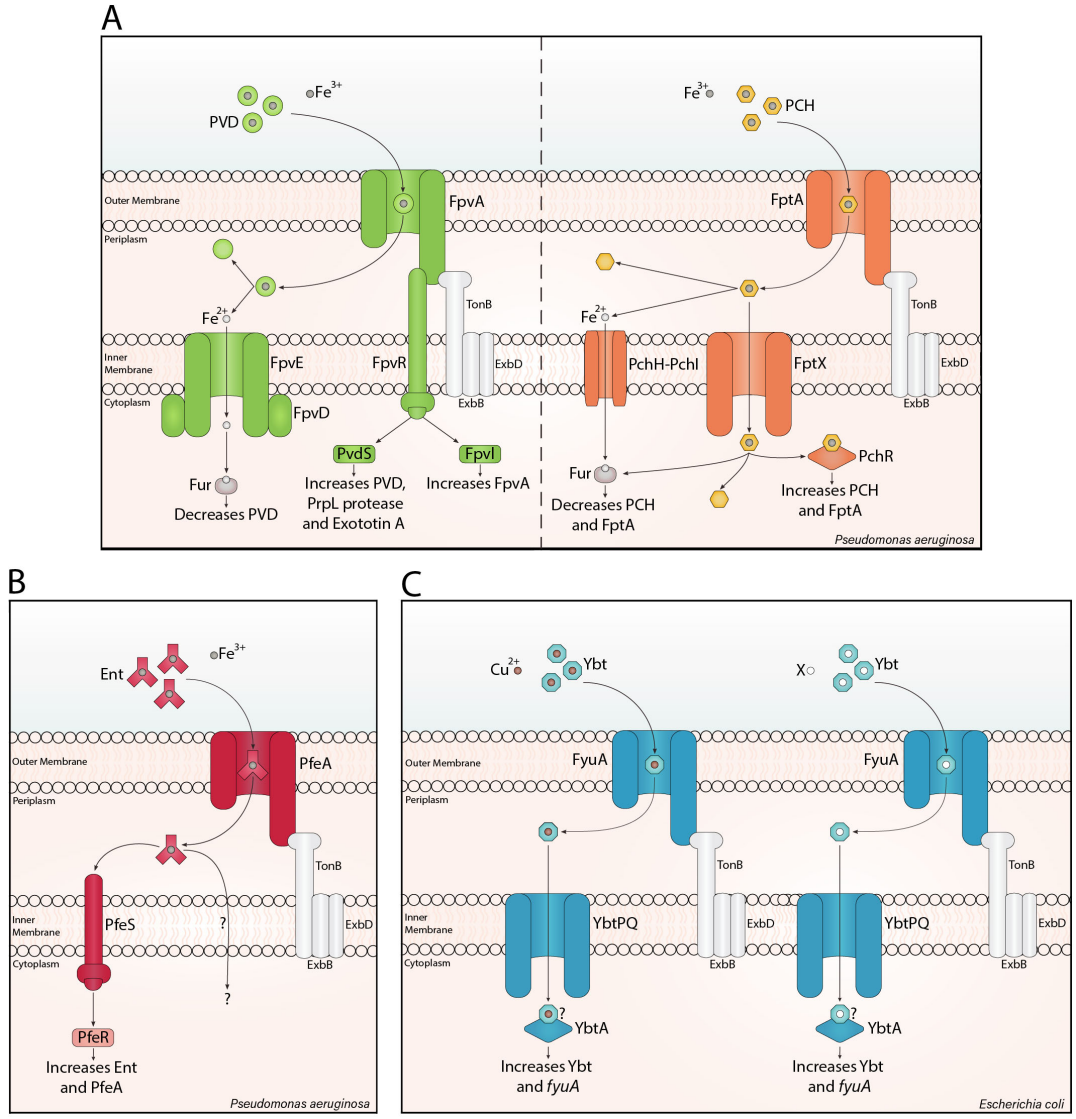
Siderophore translocation and signalling. Siderophore translocation in Gram-negative bacteria involves a siderophore-metal ion complex binding a specific β-barrel receptor in the outer membrane. Binding causes a conformational change in the receptor, translocating the loaded siderophore into the periplasm with the support of a TonB complex. The iron-loaded siderophore is typically transported through an inner membrane ATP-binding cassette transporter into the cytosol, as shown for pyochelin (PCH) and yersiniabactin (Ybt). For pyoverdine (PVD) and pyochelin, iron can be reduced in the periplasm, and the ferrous form is imported into the cytosol. **(A)** PVD signalling is mediated by FpvR, increasing PVD, virulence factors, and FpvA. PCH directly interacts with the AraC-type regulator PchR, increasing PCH and its cognate outer membrane receptor. Notably, ferrous iron inhibits PVD and PCH synthesis through the ferric uptake regulator (Fur). **(B)** In *Pseudomonas aeruginosa*, enterobactin (Ent) signalling involves PfeS, a cytoplasmic membrane-associated histidine kinase sensor, which is thought to interact with Ent (Dean et al., 1996) and trigger PfeR-mediated increase in Ent and PfeA. **(C)** Ybt-Cu^2+^ increases Ybt synthesis and *fyuA* expression through the AraC-type regulator, YbtA. The ‘?’ indicates a potential direct interaction between Ybt-X/Cu^2+^ and YbtA. Here I have added a mechanism with Ybt-X, due to the significant potential for other Ybt-metal ion mediated signalling pathways, such as Ybt-Ni^2+^, Ybt-Fe^3+^ and/or Ybt-Zn^2+^.

Siderophores can act as signalling molecules. Pyoverdine has been widely investigated as a signalling agent after being first identified by Lamont et al. (2002). When pyoverdine-Fe^3+^ binds to the outer membrane receptor FpvA, it triggers the proteolytic cleavage of the anti-sigma factor, FpvR, in the inner membrane. This releases the extracytoplasmic sigma factors, PvdS and FpvI, which induce the expression of pyoverdine (Cunliffe et al., 1995), and FpvA (Rédly and Poole, 2003) genes. Both regulators also promote the transcription of virulence factors, such as PrpL protease, exotoxin A, T3SS toxins, and haem uptake genes (**Figure 1A**; Wilderman et al., 2001; Gaines et al., 2007; Chevalier et al., 2019). Virulent *P. aeruginosa* often produce pyoverdine and pyochelin (Cox, 1982; Meyer et al., 1996). Unlike pyoverdine, pyochelin-Fe^3+^ directly binds the AraC-type regulator PchR (Heinrichs and Poole, 1993; Michel et al., 2005; Lin et al., 2013) after being imported via the outer membrane receptor FptA, and the inner membrane transporter, FptX (Cuív et al., 2004). PchR upregulates pyochelin and FptA biosynthesis (**Figure 1A**; Ankenbauer and Quan, 1994; Serino et al., 1997; Reimmann et al., 1998, 2001). Enterobactin also mediates signalling in *P. aeruginosa*. Unlike pyochelin, enterobactin-Fe^3+^ is transported into the periplasm by PfeA, where it binds to the histidine kinase PfeS, inducing autophosphorylation (Dean et al., 1996). Subsequently, this phosphoryl group is transferred to PfeR, enabling PfeR to upregulate enterobactin and PfeA (**Figure 1B**; Dean and Poole, 1993).

Pyoverdine and pyochelin signalling support efficient *P. aeruginosa* colonisation, as described by Mridha and Kümmerli (2022) in a three-phase model. When *P. aeruginosa* colonise, levels of iron stocks vary within a population; therefore, some iron stocks deplete rapidly, reducing Fur-mediated repression of siderophore synthesis. Interestingly, Fur de-repression occurs earlier for pyochelin than pyoverdine (Dumas et al., 2013), causing a difference in siderophore production. Therefore, in phase I, few cells highly produce pyochelin (Mridha and Kümmerli, 2022). In phase II, cell density and siderophore production increase, partly through siderophore-mediated self-upregulation. The increased siderophore concentrations mediate reliable signalling between cells, homogenising siderophore expression (Ross-Gillespie and Kümmerli, 2014; Mridha and Kümmerli, 2022). In phase III, pyoverdine inhibits pyochelin synthesis (Dumas et al., 2013) while increasing autoinduction and further homogeneity in the population (Mridha and Kümmerli, 2022). Here, siderophore signalling delays the production of the metabolically expensive, but efficient, pyoverdine until high cell density and low iron availability are reached, ensuring efficient iron and pyoverdine uptake by the population. Additionally, this siderophore signalling increases homogeneity, a factor that supports siderophore production (Buckling et al., 2007) and virulence (West and Buckling, 2003).

More recently, yersiniabactin has been revealed as an autoinducer. Intriguingly, Katumba et al. (2022) hypothesised that Fur-mediated regulation is inadequate to regulate the role of yersiniabactin in copper tolerance and acquisition. Subsequently, the group saw copper ions stimulate yersiniabactin synthesis, but determined that yersiniabactin-Cu^2+^, not Cu^2+^, elicits transcription of yersiniabactin and its cognate outer membrane receptor gene, *fyuA*. The AraC-type regulator, YbtA, was necessary for yersiniabactin-Cu^2+^-associated transcriptional upregulation (Katumba et al., 2022). This regulator is predicted to possess an N-terminal ligand-binding domain, similar to PchR (Fetherston et al., 1996); however, the mechanism of the yersiniabactin-Cu^2+^-YbtA interaction is unknown (**Figure 1C**).

Yersiniabactin’s involvement in copper acquisition and tolerance requires an understanding of yersiniabactin-Cu²^+^ signalling at varying copper levels. During colonisation, the host limits iron and copper. In response to low iron, Fur repression is reduced, increasing yersiniabactin production. As yersiniabactin chelates both iron and copper, Fur regulates iron and copper acquisition. Additionally, yersiniabactin-Cu²^+^ formation triggers autoinduction. In high copper conditions, increased yersiniabactin-Cu²^+^ formation enhances signalling, increasing yersiniabactin levels to sequester copper to prevent toxicity (Katumba et al., 2022). This model links the regulation of yersiniabactin for copper acquisition and tolerance.

Beyond copper, nickel increases yersiniabactin biosynthesis (Katumba et al., 2022). As yersiniabactin chelates nickel for metabolic use (Robinson et al., 2018), yersiniabactin-Ni^2+^ may act as a signalling agent.

Supporting Katumba et al. (2022); Heffernan et al. (2024) demonstrated that yersiniabactin production in early transferrin-supplemented UPEC culture is density-dependent, with delayed expression at low cell density and increased expression through (YbtA-dependent) autoinduction as cell density increases. Notably, the group did not elaborate on the mechanism of yersiniabactin autoinduction; therefore, this mechanism could be mediated by yersiniabactin-Cu^2+^, or one or more novel mechanism(s) of yersiniabactin-metal complex signalling (**Figure 1C**).

## Siderophores and reactive oxygen stress tolerance

The immune system utilises ROS against bacterial infections, but siderophores support bacterial resistance to ROS-mediated killing. For example, ROS stress increases intracellular enterobactin (Peralta et al., 2016), which protects against ROS damage (Adler et al., 2012, 2014). Interestingly, this mechanism requires the hydrolytic enzyme, Fes - suggesting enterobactin is hydrolysed to release iron and free hydroxyl groups on the catechol moieties for radical stabilisation (Povie et al., 2010; Peralta et al., 2016). This is supported by later work showing that *E. coli* strains with impaired enterobactin hydrolysis had higher ROS levels than wild type (Peralta et al., 2022). More recently, the necessity of linear enterobactin trimer dihydroxybenzoylserine (DHBS_3_) for *Salmonella enterica* serovar Typhimurium survival in extracellular peroxide was revealed (Bogomolnaya et al., 2020). The mechanism of DHBS_3_ protection was not clarified, but the known role of catechol moieties in terminating radical chain reactions hints at the potential mechanism of protection.

Interestingly, pyoverdine increases in the *P. aeruginosa* periplasm following photon- and tobramycin-induced ROS accumulation (Jin et al., 2018), preventing ROS-mediated killing. Intriguingly, *P. aeruginosa* downregulates the PvdRT-OpmQ efflux pump under photon stress to reserve pyoverdine for internal use, an event termed ‘conditional privatisation’ (Jin et al., 2018). Notably, loss of the enterobactin efflux pump in *E. coli* did not alter ROS levels compared to the wild type (Adler et al., 2014), raising the possibility of a ‘conditional privatisation’ mechanism in *E. coli*.

Pyoverdine and enterobactin upregulation in oxidative stress suggests an alternative mechanism of regulation. Notably, key regulators of ROS response mechanisms, SoxS and OxyR, regulate siderophores in *Azotobacter vinelandii* (Tindale et al., 2000), enterobactin in *E. coli* (Peralta et al., 2016) and PvdS in *P. aeruginosa* (Wei et al., 2012). However, OxyR and SoxSR activate Fur (Zheng et al., 1999), which suggests these proteins regulate siderophores through a Fur-independent mechanism in the oxidative stress response.

Unlike other siderophores, yersiniabactin-Cu^2+^ may function as a superoxide dismutase (SOD), supporting UPEC survival within macrophage-like RAW264.7 cells (Chaturvedi et al., 2014). When phagocytes engulf microbes, the phagosome NADPH oxidase catalyses the formation of superoxide anions (O_2_^•-^). As O_2_^•-^ cannot cross the membrane to mediate killing, Cu^2+^ ions are transported into the phagosome via ATP7A (White et al., 2009), where the superoxide reduces Cu^2+^ to Cu^+^. Cu^+^ ions are more freely diffusible and are directly (via iron displacement from iron-sulphur proteins), or indirectly (by reacting with H_2_O_2_ to form OH^-^ and OH^•^) toxic. Although yersiniabactin protects UPEC in macrophage-like cell phagosomes in the presence of NADPH oxidase- and Cu^2+^-derived superoxide, further experimental validation is required to confirm the SOD-like role of yersiniabactin-Cu^2+^ and its biological relevance. As phagocytes can release superoxide extracellularly (Panday et al., 2015), it would be interesting to determine whether yersiniabactin has protective qualities outside the phagosome.

Notably, neutrophils clear UPEC through copper-dependent ROS generation (Babu and Failla, 1990; Fang, 2004). Therefore, yersiniabactin has dual-function - protecting UPEC by sequestering copper to reduce ROS generation and by its SOD-like activity.

Beyond catecholate siderophores, *S. epidermidis* staphyloferrin-like siderophores detoxify ROS (Oliveira et al., 2021). However, the absence of catechol moieties makes the mechanism of this protection currently elusive.

## Siderophores and reactive oxygen stress generation

Alternatively to ROS sequestering, pyochelin has been associated with ROS production. For example, Ong et al. (2017) discovered the bacterium *Burkholderia paludis* produced pyochelin, which increased intracellular ROS, causing lipid peroxidation and cell death of *Enterococcus faecalis*.

Similarly, Gdaniec et al. (2020) saw pyochelin-enhanced ROS kill *S. aureus in vitro* when co-expressed with a high-affinity siderophore. The group proposed that, in the *S. aureus* cytosol, apo-pyochelin increases ROS by capturing Fe^3+^ produced by the Fenton reaction. However, this mechanism generates limited ROS; therefore, the mechanism of pyochelin-enhanced killing of *S. aureus* is unclear.

Interestingly, *S. aureus* possesses the staphylococcal pyochelin methyltransferase (Spm), which methylates pyochelin (on the carboxylic acid group) to reduce intracellular ROS production, increasing survival during co-infection with *P. aeruginosa* in a murine model, compared to an Spm-deficient strain (Jenul et al., 2023). Furthermore, the fungus *Phellinus noxius* (Ho et al., 2021) and soil bacterium *Bacillus amyloliquefaciens* (Molina-Santiago et al., 2021) deactivate pyochelin through the esterification of the carboxylic acid moiety.

Together, these results hint that pyochelin is used as an antimicrobial to support pathogenesis.

## Microcins

Microcins are low-molecular-weight, antimicrobial peptides made by *Enterobacteriaceae* and used as narrow-spectrum antibiotics (Baquero and Moreno, 1984). Here, we focus on class IIb microcins due to their chromosomally-encoded, C-terminal post-translational catechol-siderophore modification (Patzer et al., 2003; Vassiliadis et al., 2010).

UPEC provide an example of pathogenic bacteria that use microcins. UPEC mainly belong to phylogroup B2 (Johnson et al., 2005), which possess microcins MccH47 and MccM more often than other phylogroups (Micenková et al., 2016). The overrepresentation of microcins in UPEC is selected for due to low iron availability in urine; therefore, the high expression of siderophore receptors inadvertently increases the uptake of microcins, which allows microcin-producing strains to dominate the environmental niche.

Notably, probiotic bacteria also produce microcins to support colonisation. However, the common opportunistic pathogen nature of *Enterobacteriaceae* makes the nature of a bacterium context-dependent.

The bacteriocin nisin has been demonstrated to act as an autoinducer of its own expression (Kuipers et al., 1995). In addition, exogenous siderophores mediate autoinduction and induction of their outer membrane receptors (Ankenbauer and Quan, 1994; Guan et al., 2001). Therefore, whether the siderophore motif in microcins can regulate the production of their cognate outer membrane receptors to increase uptake is notable.

## Concluding remarks

Recently, there has been increased focus on the non-iron acquisition roles of siderophores. Notably, for progressively more siderophores the term siderophore (in Greek: sidero = iron and phore = bearer) is limiting to their range of roles. This review highlights that virulence-associated siderophores often have non-classical roles, suggesting that these roles contribute to their virulence and the producer’s pathogenesis. Furthermore, more studies have used ecologically relevant environments to explicitly show siderophores contribute to pathogenesis through non-classical mechanisms. Future research should continue using relevant environments to further understand these roles in infection. Additionally, most previous studies have focused on pyoverdine and pyochelin in *P. aeruginosa*, or yersiniabactin and enterobactin in *E. coli*. The study of more varied siderophores and siderophore-producing pathogens will elucidate more varied roles of siderophores, and their diverse contribution to pathogenesis. Finally, class IIb microcins are poorly characterised; therefore, further research in pathogens will enhance our understanding of these antimicrobials as virulence factors.
